# Invasive Candidiasis Associated with Adenovirus Pneumonia

**DOI:** 10.1155/2021/9905474

**Published:** 2021-06-05

**Authors:** Guwani Liyanage, Madhusha Gonapaladeniya, Thushari Dissanayake

**Affiliations:** ^1^Department of Pediatrics, Faculty of Medical Sciences, University of Sri Jayewardenepura, Nugegoda, Sri Lanka; ^2^Department of Microbiology, Faculty of Medical Sciences, University of Sri Jayewardenepura, Nugegoda, Sri Lanka

## Abstract

Invasive *Candida* infections in immunocompetent children lead to high morbidity and mortality despite available treatment. *Candida albicans* and *Candida parapsilosis* are the most common pathogens; however, there are newly emerging pathogenic non-*albicans* species. Adenovirus accounts for at least 5–10% of respiratory infections in children, and specific serotypes are associated with severe pneumonia. To the best of our knowledge, invasive *Candida* infection complicating adenovirus-associated pneumonia in immunocompetent children has not been reported previously. Herein, we describe a preschool child with invasive candidiasis associated with adenovirus pneumonia.

## 1. Introduction

*Candida* is a commensal of the skin and mucosa of the respiratory, gastrointestinal, and genitourinary tracts. They are true opportunistic pathogens that enter the circulation and deep tissues in high-risk individuals. Children with a critical illness are particularly vulnerable to invasive candidiasis [1]. Even though, *Candida albicans* and *Candida parapsilosis* are known to be the most common isolates, other non-*albicans* species are increasingly found [1, 2].

Adenovirus accounts for at least 5–10% of respiratory infections in children [3]. Specific serotypes are associated with severe pneumonia and prolonged illness [4]. Secondary bacterial or fungal infections could be triggered by respiratory tract erosions and neutropenia caused by adenovirus infection [4–6]. We present a case of invasive candidiasis with a non-*albicans* species in a previously healthy child associated with community-acquired adenoviral pneumonia.

## 2. Case Report

A 3-year and 6-month-old girl presented in December 2019, with fever, cough, rhinorrhea, and watery stools (a few times) for four days. Two weeks before that, she had a brief hospital stay for acute viral gastroenteritis. She was healthy and had been immunized appropriately for her age, including with BCG. The parents were nonconsanguineous and healthy. She had a high temperature (40°C) and was tachypneic, with rales predominantly on the right lung field. In addition, she had moderate dehydration on admission. After initial stabilization, oral clarithromycin and intravenous co-amoxiclav were started empirically with a presumptive diagnosis of community-acquired pneumonia.

Laboratory evaluation indicated neutropenia and thrombocytopenia (white blood cells (WBCs) = 2.78 × 10^3^/mm^3^, neutrophils = 0.83 × 10^3^/mm^3^, and platelets = 76 × 10^3^/mm^3^). The erythrocyte sedimentation rate and C-reactive protein (CRP) were 56 mm/1st h and 7.4 mg/dL. Liver enzyme levels were elevated (aspartate transaminase = 472 U/L; alanine transaminase = 194 U/L). Renal function test results were within the normal range. Antidengue IgM and IgG were negative. Chest radiography revealed peribronchial thickening, mainly in the right upper and middle zones. Polymerase chain reaction (PCR) of the nasopharyngeal aspirate was positive for adenovirus and negative for influenza and parainfluenza viruses.

Clinical improvement remained poor after 48 h of treatment. Tachypnea fluctuated between 48 and 60 breaths/min. The lowest oxygen saturation was 91%. Blood gas analysis revealed normal levels of carbon dioxide. Repeat investigations on day 8 of illness revealed reduced platelets (49 × 10^3^/mm^3^) and neutrophils (0.65 × 10^3^/mm^3^) and increased CRP levels (43 mg/dL). Repeat chest radiography revealed worsening lung condition, with interstitial infiltrates in both lung fields. No effusion was detected by ultrasound of the chest. The antibiotics were changed to intravenous cefotaxime and amikacin.

The blood culture (sampled on day 7 of illness) showed *Candida* infection. Two sputa (induced) cultures tested negative for bacteria and fungi. Cerebrospinal fluid (CSF) analysis revealed no cells and a protein concentration of 24.7 mg/dL. The CSF fungal culture was negative. *Candida* species were not isolated in urine and stool cultures. *Mycobacterium tuberculosis* was not detected in the induced sputum or gastric aspirate using the Xpert MTB/RIF assay. Echocardiography and eye assessments were normal. Abdominal ultrasound was normal.

Fluconazole was administered intravenously; however, blood cultures on illness days 9 and 11 remained positive for *Candida*. Fungal identification, which was performed using manual biochemical methods, revealed the presence of *Candida famata*. Molecular tests or gene sequencing for identification were not performed because of nonavailability of resources. With the sensitivity pattern, intravenous fluconazole was continued for an additional ten days after the first negative culture on day 13 of illness. Upon clinical recovery, complete blood count returned to normal (WBCs = 14.75 × 10^3^/mm^3^, neutrophils = 5.16 × 10^3^/mm^3^, and platelets = 896 × 10^3^/mm^3^). The levels of liver enzymes had normalized, and chest radiography on day 18 indicated recovery. The subsequent immunological workup revealed parameters within the normal range (immunoglobulin levels, lymphocyte subsets, and neutrophil function tests). The serum tested negative for HIV antigen and antibody. The patient remained well, without signs of relapse during the subsequent follow-up.

## 3. Discussion

Invasive candidiasis has not been reported in immunocompetent children with adenoviral infection. Although it is difficult to establish a causal relationship between the adenoviral and *Candida* infections, it is tempting to speculate that adenovirus infection was a predisposing factor for candidemia in this patient for two reasons. First, invasive *Candida* infections associated with other respiratory viruses have been reported [7]. For example, candidemia has been observed in patients critically ill with COVID-19 [8, 9]. Second, the entry point of *Candida* into the bloodstream could have been through the respiratory tract erosions caused by severe adenoviral infection. As repeated sputa cultures were negative for *Candida* in our patient, alternatively, the pathogens may have invaded through sites other than the respiratory tract. *Candida* spp. have historically been considered commensals of normal human microbiota of the oral cavity, skin, and gastrointestinal tract [10]. This healthy microbiota is often altered in disease states [10]. Broad-spectrum antibiotic treatment and viral-induced neutropenia may have contributed to invasive candidiasis in our patient [11]. Viral infections frequently cause neutropenia owing to bone marrow suppression and peripheral destruction [12].

*Candida* is the third most common healthcare-associated bloodstream infection [13]. Colonization is the first step to invasive disease. They exploit host vulnerabilities to enter the circulation and deep tissues, thus behaving like a true opportunistic pathogen. Systemic candidiasis is divided into two primary syndromes: candidemia and disseminated candidiasis (defined as a *Candida* infection in sterile target organs, with or without positive blood culture results). We assumed that our patient had candidemia as we could not detect *Candida* infection in other organs.

*Candida albicans* are responsible for at least one-third of the isolates [2]. *Candida parapsilosis* is the second most common [2, 13, 14]. Opportunistic infection with *Candida famata* is rare [15]. A few previous case reports of *Candida famata* fungemia in immunocompetent adults and children had been described [15, 16]. However, most reported cases occurred in immunocompromised or burn patients [17, 18].

The absence of specific clinical features delays the diagnosis [5]. Conventionally, the diagnosis of candidemia is based on blood cultures. However, the sensitivity of blood culture is between 63% and 86% [19]. This is even worse in children due to low-volume samples. Moreover, it can take days to turn positive and even longer to identify the species. Nonculture-based methods such as DNA detection by PCR have been studied to assist early diagnosis [20]. *Candida famata* was identified in our patient using biochemical methods. However, misidentification of certain *Candida* species by biochemical methods has been reported [21, 22]. Although molecular techniques based on DNA or gene sequencing are helpful for accurate identification, these methods are less available in resource-poor settings. Accurate identification is important for species-oriented treatment as the newly emerging pathogens may differ significantly in their antifungal susceptibilities [22]. In vitro studies have demonstrated increased susceptibility of *Candida famata* to amphotericin B compared to fluconazole [17]. However, according to many reports, fluconazole monotherapy had been effective in *Candida famata* fungemia [16].

This report highlights that invasive candidiasis is a complication of severe community-acquired pneumonia. Active surveillance for invasive candidiasis in critically ill children is essential for the timely diagnosis of this severe opportunistic infection. Less expensive, rapid diagnostic methods should be further explored for early diagnosis and species-oriented treatment.

## Data Availability

The data used to support the findings of this study are available from the corresponding author upon request.
